# Septic Pulmonary Emboli or Pulmonary Metastasis in a Patient with Diabetes Mellitus?

**DOI:** 10.3390/jcm7070176

**Published:** 2018-07-14

**Authors:** Meng-Yu Wu, Ling-Chi Lee, Yu-Long Chen, Yung-Hsiang Yeh, Chia-Jung Li, Giou-Teng Yiang

**Affiliations:** 1 Department of Emergency Medicine, Taipei Tzu Chi Hospital, Buddhist Tzu Chi Medical Foundation, New Taipei 231, Taiwan; skyshangrila@gmail.com (M.-Y.W.); nakahikari@gmail.com (L.-C.L.); yulong0129@gmail.com (Y.-L.C.); 2 Department of Emergency Medicine, School of Medicine, Tzu Chi University, Hualien 970, Taiwan; 3 Division of Gastroenterology, Department of Internal Medicine, Chang Bing Show Chwan Memorial Hospital, Changhua 500, Taiwan; 4 Research Assistant Center, Show Chwan Memorial Hospital, Changhua 500, Taiwan

**Keywords:** emphysematous pyelonephritis, gas accumulation, septic pulmonary emboli

## Abstract

Emphysematous pyelonephritis is a rare but life-threatening infection characterized by an accumulation of gas in the renal parenchyma. A high mortality rate was reported, and timely administration of broad-spectrum antibiotics for enteric gram-negative bacilli, such as *Escherichia coli*, *Enterobacter*, and *Klebsiella pneumoniae*, was recommended for improving the clinical outcome. Computed tomography is a useful tool for identifying gas accumulation within the perirenal space. Abdominal ultrasound and abdominal plain film are alternative options with lower accuracy rates. Here, we present the case of a 49-year-old male patient who presented with acute-onset progressive abdominal cramping pain and dysuria. Diffuse bilateral opacities over the lung field and a heterogeneous mass with air density in the abdomen were found on radiological examination. Multiple septic pulmonary emboli and emphysematous pyelonephritis were diagnosed using computed tomography. After emergency percutaneous nephrostomy and administration of broad-spectrum antibiotics, the patient was discharged in a stable condition and followed up at the outpatient department. This report also describes the clinical and imaging features of emphysematous pyelonephritis and highlights that early diagnosis and timely administration of broad-spectrum antibiotics may help avoid a potentially devastating outcome.

## 1. Introduction

Emphysematous pyelonephritis (EPN) is a rare but life-threatening infection with a mortality rate of up to 50% [1]. The typical imaging feature is an accumulation of gas in the renal parenchyma. The timely administration of broad-spectrum antibiotics for enteric gram-negative bacilli, such as *Escherichia coli*, *Enterobacter*, and *Klebsiella pneumoniae*, can significantly improve the outcome. Here, we present the case of a 49-year-old male patient who presented with acute-onset progressive abdominal cramping pain and dysuria. Diffuse bilateral opacities over the lung field and a heterogeneous mass with air density in the abdomen were found on radiographic examination. Multiple septic pulmonary emboli and EPN were diagnosed using computed tomography (CT). After emergency percutaneous nephrostomy and administration of broad-spectrum antibiotics, the patient was discharged in a stable condition and followed up at the outpatient department. This report also describes the clinical and imaging features of EPN and highlights that early diagnosis and timely treatment may help avoid a potentially devastating outcome.

## 2. Case Presentation

**Figure 1:**
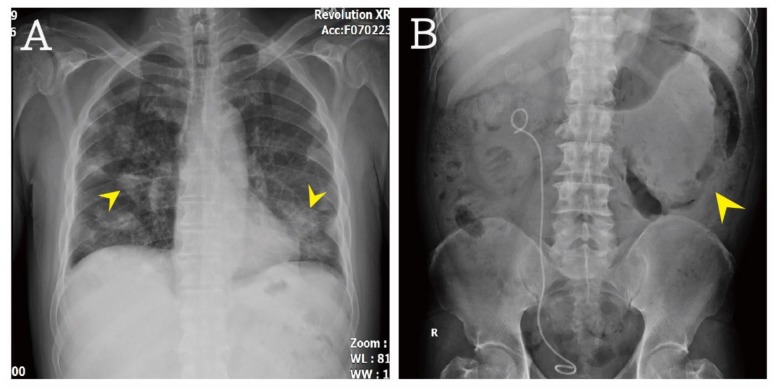
(**A**): Multiple opacities over bilateral lung field were noted (arrow head). (**B**): A heterogeneous mass with air density at left side (arrow head) was found.

A 49-year-old male patient presented with acute-onset progressive abdominal cramping pain that had started 1 day previously. He had a medical history of poorly controlled diabetes mellitus and hypertension, as well as renal stone formation after percutaneous nephrolithotomy with double J replacement. There was no history of trauma. Dysuria and mild urgency were noted. He denied having any fever, chills, cough, chest pain, nausea, vomiting, and diarrhea. His temperature was 36.8 °C, blood pressure was 162/89 mmHg, and heart rate was 131/min. On physical examination, a hyperactive bowel sound was noted, accompanied by whole abdominal tenderness, especially at the left quadrant. The Murphy sign was negative, and no tenderness was noted at McBurney’s point. There was no bilateral knocking pain. The laboratory results were as follows: white blood cell count 40,250/µL (band-form neutrophils 2.0%, segment-form neutrophils 86.0%, lymphocytes 5.0%, eosinophils 0.0%, and monocytes 6.0%), hemoglobin 6.7 g/dL, platelet count 645,000/mL, blood urine nitrogen 51 mg/dL, creatinine 1.9 mg/dL, sodium 124 mmol/L, potassium 5.5 mmol/L, glucose 790 mg/dL, alanine aminotransferase 19 U/L, lipase 768 IU/L, total bilirubin 1.00 mg/dL, troponin I <0.01 μg/L, ketone bodies 4.5 mmol/L, and serum osmolarity 336 mOsm/kg. The urinalysis results were as follows: Red blood cell count 10–19/high-power field (HPF), white blood cell count 10–19/HPF, glucose 4+, ketone bodies 1+, bacteria 1+/HPF, and yeast 3+/HPF. The venous blood gas analysis revealed the following results: pH 7.390, pCO_2_ 29.2 mmHg, pO_2_ 44.5 mmHg, HCO_3_ 17.3 mmol/L, actual base excess −6.3 mmol/L, base excess in extracellular fluid −7.6 mmol/L, and O_2_ saturation 78.9%. The detailed results of laboratory evaluations are listed in Table 1. The plain film showed diffuse bilateral opacities over the lung field (Figure 1A). The kidney, ureter, and bladder (KUB) study revealed gallbladder stone, double J catheter placement from the pelvic cavity to the right renal region, and a heterogeneous mass with air density at the left side (Figure 1B).

**Figure 2:**
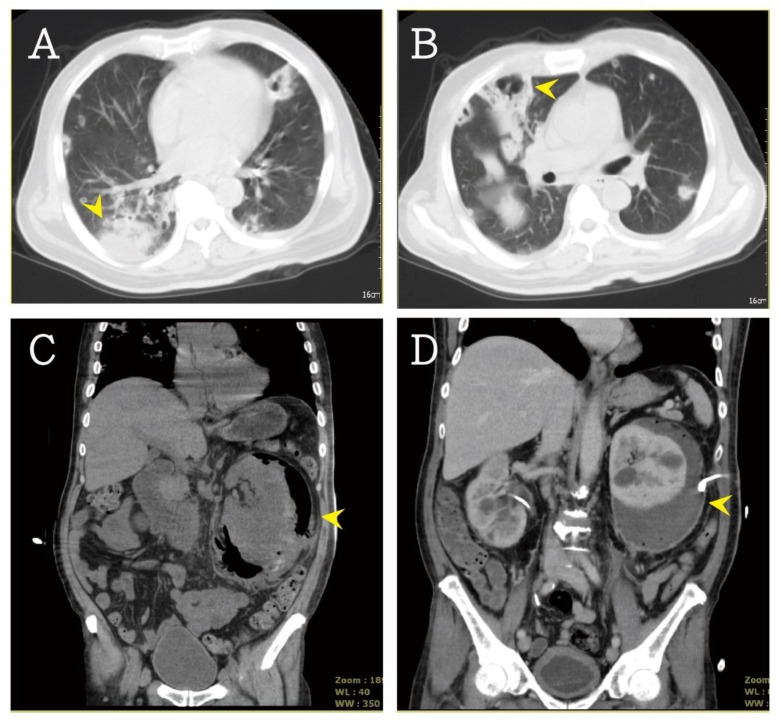
(**A,B**) Diffuse bilateral nodular densities with poor margin and cavitation was noted (arrow head) in CT scan. (**C**) An enlarged kidney, destroyed renal parenchyma with fluid and gas collections in renal capsule, was found (arrow head). (**D**) After emergency drainage (arrow head), the fluid accumulation was improved.

**Figure 3:**
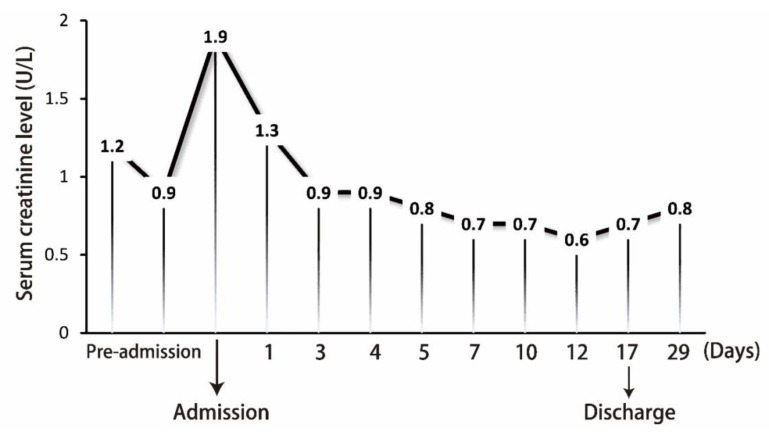
The follow-up serum creatinine level from the admission to discharge.

Contrast-enhanced abdominal CT was performed, which revealed several cavitary nodules in both lower lung fields (Figure 2A,B). A perirenal heterogeneous mass with gas density was found inside the left renal capsule (Figure 2C). According to the above-mentioned clinical symptoms and images, EPN and multiple septic pulmonary emboli were suspected. The broad-spectrum antibiotics meropenem and teicoplanin were administrated for sepsis. Meropenem was administered for 13 days and teicoplanin was used for 9 days; then, the patient was shifted to ceftriaxone for 3 days. In addition, the patient’s hyperkalemia was treated with insulin in 5% glucose solution and calcium polystyrene sulfonate powder (Kalimate). Transfusion of 2 U packed red blood cells was done for anemia. Famotidine was administered to prevent stress ulcer. Emergency percutaneous nephrostomy was done (Figure 2D), and pus and urine were collected for culture. The cultures showed *E. coli* growth in urine and pus. No growth of significant aerobic or anaerobic pathogens in blood was noted. After timely treatment, the sepsis was controlled. The follow-up plain film and abdominal CT revealed a few cavitary nodules and less accumulation of perirenal abscess. The patient was discharged and followed up at the urologic and chest outpatient departments. The follow-up serum creatinine level is summarized in Figure 3. This study was approved by the Institutional Review Board (IRB) of Taipei Tzu Chi Hospital, Buddhist Tzu Chi Medical Foundation (IRB no. 07-CR-059).

## 3. Discussion

EPN is a life-threatening and fulminant infection disease, characterized by the formation of gas within the kidney. The progression of EPN to sepsis is rapid in the absence of timely therapeutic interventions. EPN is not a common condition. In 2009, Pontin and Barnes [2] reported that there have been 52 patients with EPN during the last 32 years. Diabetes mellitus is a significant risk factor for EPN. In their report, diabetes mellitus accounted for up to 90% of EPN cases. Middle-aged female patients with diabetes comprise the main population of EPN. The typical symptoms of EPN include fever, dysuria, and flank pain. Tsu et al. [3] reported that the mean duration of symptoms before admission was 4.7 ± 3.1 days and that fever was the most common symptom, followed by loin pain (33.3%), hematuria (8.3%), and palpable mass (16.7%). However, EPN-induced septic emboli involving the distant organs have been rarely reported. In 2014, Yadav et al. [4] reported on a 67-year-old male patient who presented with air in the left renal vein and collecting system of the left kidney with multiple septic emboli in the lung field. A similar case was reported by Elvas et al. [5], with cavitated damage in the lungs and suspected probable septic embolism. The accurate incidence rate of EPN with pulmonary involvement is not clear owing to the rarity of published cases. In our case, the patient presented with flank pain and multiple medical problems, including uncontrolled hyperglycemia, acidosis, and electrolyte imbalance. Similar results were reported by Joseph et al. [6]. To reach a diagnosis of EPN, imaging studies such as ultrasonography, KUB, and CT are rapid and useful as a first step to evaluate the kidney. In previous studies, the accuracy rate of ultrasonography ranged from 50% to 86% and the diagnosis rate of CT for EPN was about 100% [2,7,8]. Nowadays, CT is recommended as the first-choice imaging modality to diagnose EPN. In our case, severe accumulation of gas within the perirenal space made the renal border well defined and enabled the diagnosis using a KUB study. In the early stage of EPN, the less formation of gas may lead to a missed diagnosis of EPN.

According to the imaging features on CT, Wan et al. [7] reported a classification that divides EPN into 2 types that strongly correlated with prognosis: type I EPN, characterized by parenchymal destruction without fluid or gas collection and has a mortality rate of about 69%; type II EPN, characterized by fluid collections with bubbly gas and has a mortality rate of about 18%. Huang and Tseng [9] provided another classification in 2000: Class I, only accumulation of gas in the collecting system; class II, only parenchymal gas; class IIIa, gas into the perirenal space; class IIIb, gas into the pararenal space; and class IV, bilateral EPN or EPN in a solitary kidney. In class I and II EPN, medical therapy alone or with percutaneous drainage was suggested as the first step of treatment. In class IIIa and class IIIb EPN, the failure rate of combined percutaneous drainage was up to 30–71%. In class VI EPN, surgical intervention was suggested to control the infectious condition. In our patient, class IV EPN was diagnosed using CT (Figure 2C). Emergency percutaneous nephrostomy was done to lower the mortality risk.

The current analysis of pathogens in patients with EPN revealed that *E. coli* is the most common pathogen (accounting for 43.6%), followed by *Proteus mirabilis* (15.6%), *Pseudomonas aeruginosa* (12.5%), *Enterococcus* species (12.5%), *K. pneumoniae* (9.4%), and *Candida species* (9.4%) [10]. Empiric antibiotic treatment was suggested based on local antibiograms and primarily targeted *E. coli*, *P. mirabilis*, *P. aeruginosa*, *Enterococcus* species, and *K. pneumoniae*. The single-agent options effective against EPN are at least third-generation cephalosporins and carbapenems. For high-risk patients, the combination of amikacin and third-generation cephalosporin is an option [11]. In this case, report, we highlighted that the clinical and imaging features of EPN may be helpful for physicians to diagnose EPN early, and to provide timely medical and surgical intervention for better outcomes.

## Figures and Tables

**Table 1:** The laboratory evaluation in the patient.

Variables	Patient Data	Normal Range	Variables	Patient Data	Normal Range
White cell count	40,250/µL	3500–11,000/µL	Ketone body	4.5 mmole/L	<0.6 mmole/L
Band-form neutrophils	2.0%	0–3%	Serum osmolarity	336 mOsm/Kg	280–295 mOsm/Kg
Segment-form neutrophils	86.0%	45–70%	Venous blood gas		
Lymphocytes	5.0%	25–40%	pH value	7.390	7.31–7.41
Eosinophils	0.0%	1–3%	pCO2	29.2 mmHg	41–51 mmHg
Monocytes	6.0%	2–8%	pO2	44.5 mmHg	80–100 mmHg
Hemoglobin	6.7 g/dL	12–16 g/dL	HCO3	17.3 mmole/L	22–26 mmole/L
Platelet counts	645,000/μL	150,000–400,000/ μL	ABE	–6.3 mmole/L	–3.3–2.3 mmole/L
Blood urine nitrogen (BUN)	51 mg/dL	7–18 mg/dL	BEecf	–7.6 mmole/L	–
Creatinine	1.9 mg/dL	0.55–1.02 mg/dL	O2 saturation	78.9%	–
Sodium	124 mmole/L	136–145 mmole/L	Urinalysis		
Potassium	5.5 mmole/L	3.5–5.1 mmole/L	Red cell count	10–19/HPF	0–2/HPF
Glucose	790 mg/dL	70–100 mg/dL	White cell count	10–19/HPF	0–5/HPF
Alanine aminotransferase	19 U/L	14–59 U/L	Glucose	4+	−/+
Lipase	768 IU/L	73–393 IU/L	Ketone body	1+	–/+
Total bilirubin	1.00 mg/dL	0.0–1.0 mg/dL	Bacteria	1+/HPF	0/HPF
Troponin I	<0.01 μg/L	<0.01 μg/L	Yeast	3+/HPF	0/HPF
CRP	30.03 mg/dL	<0.33 mg/dL			
